# Mycoplasmosis in Poultry: An Evaluation of Diagnostic Schemes and Molecular Analysis of Egyptian *Mycoplasma gallisepticum* Strains

**DOI:** 10.3390/pathogens12091131

**Published:** 2023-09-05

**Authors:** Ahmed Al-baqir, Ola Hassanin, Mohammed Al-Rasheed, Mohamed S. Ahmed, Mahmoud H. A. Mohamed, Mohamed Shawky El Sayed, Mohamed Megahed, Azza El-Demerdash, Youserya Hashem, Amal Eid

**Affiliations:** 1 Avian and Rabbit Medicine Department, Faculty of Veterinary Medicine, Zagazig University, Zagazig 44511, Sharkia, Egypt; amelbaqr44@gmail.com (A.A.-b.);; 2 Department of Clinical Sciences, College of Veterinary Medicine, King Faisal University, P.O. Box 400, Al-Ahsa 31982, Saudi Arabiaolahassanin@zu.edu.eg (M.S.A.); mahmoudhassanain@yahoo.com (M.H.A.M.); 3 Avian Research Center, King Faisal University, P.O. Box 400, Al-Ahsa 31982, Saudi Arabia; mabdelmoaty@kfu.edu.sa; 4 Department of Poultry Diseases, Faculty of Veterinary Medicine, South Valley University, Qena 83523, Egypt; 5 Veterinary Serum and Vaccine Research Institute, Abassia, Cairo 11381, Egypt; 6 Laboratory of Biotechnology, Department of Microbiology, Agriculture Research Centre (ARC), Animal Health Research Institute (AHRI), Zagazig 44516, Egypt; dr.azzasalah@yahoo.com; 7 Mycoplasma Department, Animal Health Research Institute, Agricultural Research Center, Dokki, Giza 12618, Egypt; ramyelkomy_78@yahoo.co.uk

**Keywords:** *Mycoplasma gallisepticum*, qRT-PCR, *mgc2*, *atpG*, *mraW*, *ugpA*, *DUF31196*, *lgT*

## Abstract

Infections with *Mycoplasma gallisepticum* (MG) in poultry are associated with a wide range of disease conditions, including those affecting the respiratory and reproductive systems. The purpose of this study was to endorse the more sensitive diagnostic scheme for MG infection and identify the best molecular marker for MG phylogenetic analysis using six housekeeping genes: *mgc2*, *mraW*, *atpG*, *ugpA*, *DUF31196,* and *lgT*. For these purposes, 55 poultry flocks of different species were screened using either qRT-PCR or PCR techniques analogous to conventional culturing from non-cultured and cultured swabs on PPLO broth. The rate of MG positivity was the highest when using qRT-PCR from cultured broth (89.0%) and the lowest when using conventional culturing (34.5%). Compared to qRT-PCR from broth, statistical analysis using the Roc curve in MedCalc statistical software showed that the PCR schemes (qRT-PCR from swabs and PCR from swabs and broth) performed better than conventional culturing in terms of sensitivity, accuracy, and area under the curve (AUC), suggesting that they may be more reliable schemes. Further support was added by Cohen’s kappa test, showing moderate agreement between the molecular approaches. Among the six screened genes, *mgc2* and *mraW* had the highest detection rates (69% and 65.4%, respectively). The comparative phylogenetic analysis revealed that *mgc2* or *atpG* gene sequences distinguished MG isolates into different clades with high discriminatory power.

## 1. Introduction

The poultry production industry is a rapidly expanding business worldwide. Mycoplasma is one of the most economically detrimental diseases affecting the poultry production sector [1]. It is caused by *Mycoplasma gallisepticum* (MG), which belongs to the class Mollicutes, the order Mycoplasmatales, and the family Mycoplasmataceae [2]. It is the smallest self-replicating cell with a diameter of 0.15–0.3 μm. Mycoplasma can pass through the 0.22 and 0.1 µm sterilizing-grade filters that are used to prepare cell culture media components [3]. MG has the potential to affect a wide variety of avian species, including chickens, turkeys, house finches, songbirds, game birds, peafowl, Japanese quail, bobwhite quail, pigeons, ducks, and geese [4,5]. It substantially harms the poultry industry through decreased egg production, poor growth rate, low feed conversion efficiency, degraded carcass quality, increased mortality, and high costs for medication and vaccination [6,7]. Additionally, it affects the usefulness of chicken viral vaccines, and it has a synergistic effect with other viral pathogens caused by respiratory diseases such as infectious bronchitis virus (IBV), Newcastle disease virus (NDV), and avian metapneumovirus [8,9]. MG is the primary pathogen responsible for chronic respiratory disease (CRD) in chickens and infectious sinusitis in turkeys, chickens, game birds (including quail), pigeons, and passerine birds of all ages [10]. Clinical signs of MG in poultry range from subclinical to obvious respiratory symptoms such as coughing, sneezing, conjunctivitis, nasal exudate, and respiratory rales [2]. Several earlier reports were concerned with the enhancement and validation of different Mycoplasma species’ diagnostic schemes all over the world [11,12]. Since the organism can exist in a subclinical form, sensitive, specific, accurate, and time-saving diagnostic tools are critical for disease control. The culture method was considered the gold standard for mycoplasma diagnosis. Additional discriminatory digitonin biotyping was necessary to distinguish between Mycoplasma and Acholeplasma, according to sterol requirement criteria of mycoplasma growth [13]. However, due to the fastidious nature of MG, culturing can take up to 3 weeks to detect obvious growth [12]. Further, the overgrowth of saprophytic mycoplasmas and other bacterial pathogens is another common issue with cultivation procedures [1]. As a result, the development of advanced diagnostic methods has a significant impact on the diagnosis of such infections, producing results significantly faster than the culturing method. Molecular diagnostic tools are currently being used as alternatives to traditional cultivation methods, with high specificity and sensitivity outputs [14]. Competent epidemiological investigation tools are also required to distinguish between vaccines and field MG strains in suspected flocks [15,16]. The aforementioned can be achieved via the selection of proper molecular targets that can be detected directly and discriminated between the different strains according to their virulence or host-specific origin [17,18]. The gene-targeted sequencing (GTS) analysis of MG surface proteins, such as the *gapA*, *mgc2*, *pvpA,* and *MGA_0319*, was developed by Ferguson et al. [15]. However, there are few studies and publications dealing with the molecular characterization and sequencing of MG isolates from species aside from chickens. The current study thought to validate various diagnostic schemes in order to identify a more sensitive and specific system for MG diagnosis in different host species. Furthermore, for six housekeeping genes, a broader molecular data set for different MG isolates with different host origins was provided.

## 2. Materials and Methods

### 2.1. Animal Ethics

The Animal Welfare and Research Ethics Committee, Faculty of Veterinary Medicine, Zagazig University, Egypt, established the guidelines for this study, which received ZU-IACUC4342022 approval. All bird-handling procedures were followed in accordance with the applicable guidelines and rules. The biological wastes were disposed of in accordance with the regulations in place.

### 2.2. Clinical and Postmortem Examination

During the period 2019–2022, 55 non-vaccinated poultry flocks suspected of being MG infected were investigated in 6 Egyptian governorates (Sharkia, Dakahlia, Ismailia, Damietta, Port-Said, and South Sinai). Three hundred thirty birds (six from each flock) were examined clinically, representing various species and breeds such as turkey Poults, broiler chickens, chicken layers, chicken breeders, and quail broilers and breeders. Their descriptive data are summarized in Table S1a–e. All pathological changes were recorded and analyzed during the clinical and postmortem examinations according to the work by Zhang et al. [19].

### 2.3. Sampling

**Figure 1:**
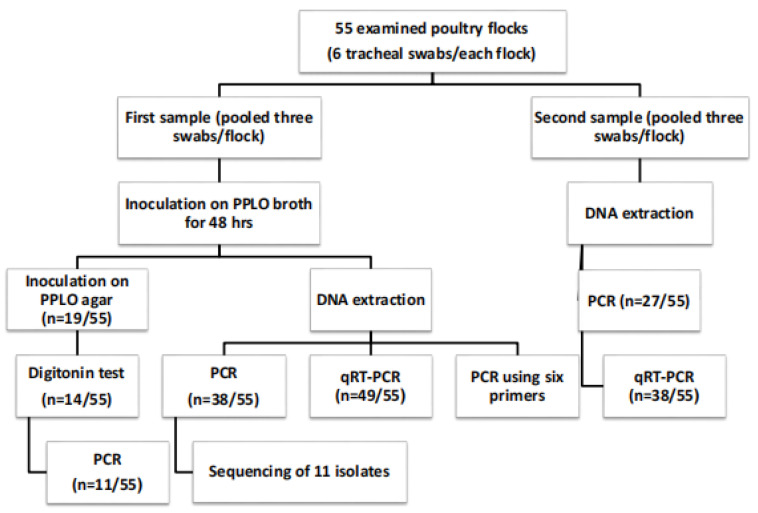
Flowchart of the study design and numbers of flocks analyzed.

Six tracheal swabs were collected from each flock under aseptic conditions and then placed in tubes with 1 mL of avian Mycoplasma broth medium (Tryptone 1.7 gm, Sodium Chloride 0.5 gm, Soya Peptone 0.3 gm, Di-potassium Phosphate 0.25 gm, Glucose 0.25 gm and Bovine Albumin 0.5 gm per 100 mL). The medium was supplied by the Mycoplasma Department, Animal Health Research Institute, Dokki, Giza, Egypt, and stored in special sterile ice-filled containers to prevent swabs from drying out after sampling [20]. Swabs were pooled and divided into two groups of three (3/each). The first set was used for culture and subsequent molecular studies, while the second was tested directly at the molecular level. Below is a flowchart of the study design and numbers of flocks analyzed (Figure 1).

### 2.4. Microbiological Characterization of MG Isolates

The first set of swabs underwent bacterial isolation [21] followed by bio-typing with a digitonin test [22] to differentiate between Mycoplasma and Acholeplasma isolates. Following inoculation into pleuropneumonia-like organisms (PPLO), broth (BD Difco™, SLS, Dublin, Ireland) with components (PPLO broth base, 14.7 gm, Glucose, 10 gm, horse serum, 150 mL, fresh yeast extracts, 100 mL, phenol red 1%, 2.5 mL, thallium acetate 10%, 2.5 mL, penicillin G potassium, 10 units, distilled water, 1000 mL) was incubated at 37 °C with 10% carbon dioxide (CO_2_) for 48 h. Daily observations were made on the PPLO broth to observe the color change. However, the color change was not considered as a positive result but only indicator. Hence, both positive and negative color changed PPLO broth were subjected for further identification. Loopful from all incubated PPLO broth were then streaked on a PPLO agar (PPLO agar base, 10.5 gm, yeast extracts 5%, 50 mL, thallium acetate 2%, 25 mL, penicillin G sodium, 500,000 IU, sterile horse serum, 75 mL, DNA 0.2%, 5 mL, and distilled water, 500 mL) and incubated at 37 °C with 10% CO_2_. The PPLO agar plates were checked every day under a dissecting microscope for the characteristic fried egg appearance of Mycoplasma colonies for up to 20 days. To distinguish between Mycoplasma and Acholeplasma isolates, the characteristic colonies that had been purified underwent a digitonin sensitivity test. The purified colonies were then stored as agar blocks at −20 °C.

### 2.5. Molecular Characterization of MG Isolates

#### 2.5.1. DNA Extraction

DNA was extracted from three different sets of samples: swabs cultured on PPLO broth after 48 h of incubation, non-cultured swabs, and pure colonies with characteristic morphology on a PPLO agar. DNA extraction was carried out in accordance with the instructions provided by the manufacturer of the QIAamp DNA Mini kits (Qiagen, Hilden, Germany). Thereafter, the DNA was stored at −20 °C for further molecular analysis (PCR and qRT-PCR).

#### 2.5.2. PCR Screening of the mgc2 Gene

PCR was conducted in a 25 μL reaction volume containing 12.5 μL One PCR™ Hot Star (GeneDirex, Taiwan, China), 1 μL of each of the forward primer and the reverse primer (Table 1), 5 μL of the template DNA, and 5.5 μL of nuclease-free water. The reaction was performed in an applied bio system 2720 thermal cycler under the following cycling conditions: 94 °C for 3 min followed by 40 cycles of initial denaturation at 94 °C for 20 s, primer annealing at 56 °C for 40 s, primer extension at 72 °C for 60 s, and final extension at 72 °C for 7 min. The amplified PCR products were electrophoresed in 1% agarose gel at 110 V for 20 min and documented using an ultraviolet trans-illuminator after ethidium bromide staining.

#### 2.5.3. QRT-PCR Screening of the mgc2 Gene

A Step One Plus^TM^ apparatus was used to perform the amplifications (Applied Bio systems, Foster City, CA, USA) in the Biotechnology Laboratory, Animal Health Research Institute, Zagazig Branch, Egypt, according to [25]. As a template, 5 μL of extracted DNA was mixed with 15 μL of master mix (iQ™ SYBR Green Supermix; Bio-Rad, Hercules, CA, USA). The master mix contained 10 μL of iQ™ SYBR Green Supermix, 1 μL each of forward and reverse primers (Table 1), and 3 μL of deionized water. The cycling parameters were as follows: an initial denaturing step of 95 °C for 3 min followed by 40 cycles of 95 °C for 30 s and annealing/extension at 60 °C for 60 s. The fluorescence data were collected at the exponential phase of the reaction, and a cycle threshold (Ct) of less than 35 and PCR products with a melting temperature of 81.55 °C were considered positive. Melting curve analyses were performed immediately after the reaction to confirm the specificity of the PCR product. Each reaction had negative (PCR master mix without DNA template) and positive controls for MG, which were obtained from Thermo Fisher Specialty Diagnostics Ltd., Hampshire, the UK, and are available at https://thermofisher.com/microbiology, accessed on 5 August 2023.

### 2.6. Statistical Analysis

The data were analyzed using Microsoft Excel (Microsoft Corporation, Redmond, WA, USA). The Roc curve of MedCalc statistical software was used for assessing the sensitivity, specificity, accuracy, positive prediction values, and negative prediction values of different diagnostic schemes. Cohen’s kappa test was used to test the quality of agreement between tests. The scale used to interpret the κ estimates was as follows: ≤0, no agreement; 0.01–0.20, slight agreement; 0.21–0.40, fair agreement; 0.41–0.60, moderate agreement; 0.61–0.80, substantial agreement; and 0.81–1.00, almost perfect agreement. Statistical significance was determined as *p* < 0.05.

### 2.7. PCR Amplification of Six Housekeeping Genes

For all of the flocks examined, PCR was performed on DNA samples extracted from PPLO broth that had been incubated for 48 h. PCR was carried out in a 25 μL reaction volume containing 12.5 μL One PCR™ Hot Star (GeneDirex, China), 20 pmol of each of the forward and the reverse primer, 5 μL of the template DNA, and 5.5 μL of nuclease-free water. The reaction was performed in an applied biosystem 2720 thermal cycler under the following cycling conditions: 94 °C for 5 min followed by 35 cycles of initial denaturation at 94 °C for 40 s, primer annealing at 56 °C for 40 s, primer extension at 72 °C for 60 s, and final extension at 72 °C for 5 min. The amplified PCR products were electrophoresed in 1% agarose gel at 110 V for 20 min and documented using an ultraviolet trans-illuminator after ethidium bromide staining.

### 2.8. Sequencing and Phylogenetic Analysis

Six gene loci were targeted for genomic sequencing (*mgc2*, *ugpA*, *mraW*, *atpG*, *DUF31196,* and *lgT*). Thirty-five purified PCR products of eleven isolates representing the positively affected flocks were submitted for genomic sequencing, including (broiler chickens n = 3, turkey Poults n = 3, chicken layers n = 3, and quail breeders n = 2). The sequencing was performed by the SolGent (Solution for Genetics Technologies) company on an Applied BiosystemsABI3730XLDNA (850 Lincoln Centre Drive Foster City, CA 94404, USA). Purified products were sequenced in both in the forward and reverse directions using a ready reaction Big dye Terminator V3.1 cycle sequencing kit (Perkin-Elmer/Applied Bio systems, Foster City, CA, USA). To determine sequence identity, BLAST^®^ analyses were run on the obtained sequences against previously published sequences in GenBank (http://www.ncbi.nlm.nih.gov/BLAST, accessed on 5 August 2023). For the comparative analysis of the sequences, the CLUSTALW multiple sequence alignment program, version 1.83 of the MegAlign module of Laser gene DNA Star software pairwise designed by [26], was used. Phylogenetic trees were constructed by the maximum likelihood method, and genetic relations among MG strains were concluded via a Jukes–Cantor model. The rate of variation among the sites was described by a gamma distribution, and 1000 bootstrap replicates were calculated using MEGA version 6. The six phylogenetic trees were rooted with the F vaccine strain.

## 3. Results

### 3.1. Clinical Signs

The 55 examined flocks showed clinical signs such as ruffled feathers, decreased feed and water intake, depression, sneezing, coughing, and diarrhea. In addition, mortality rates ranging from 3 to 20% were observed in the examined broiler chickens. Severe respiratory signs such as nasal and ocular discharge, gasping, and head swelling were recorded. Lameness and nervous signs were observed in two broiler chicken flocks. Drops in egg production ranging from 6 to 43%, mild respiratory signs (sneezing and coughing), and mortality (1–4%) were observed in the investigated layer chicken flocks. Along with respiratory distress, the most common clinical findings in turkey Poults were unilateral or bilateral infraorbital sinus swelling, with mortality ranging from 4 to 25%. Quail breeders experienced a drop in egg production (10–30%), decreased hatchability, mortality (2–5%), and mild respiratory symptoms.

### 3.2. Postmortem Lesions

**Figure 2:**
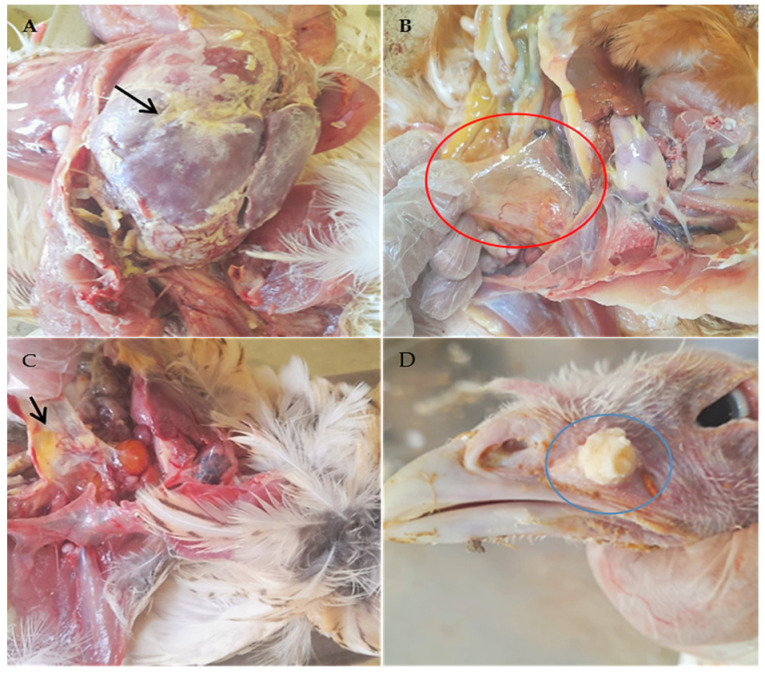
Postmortem lesions of the investigated poultry flocks: (**A**) a Hubbard broiler chicken, 29 days of age (flock 8-B) with Fibrinous perihepatitis (black arrow), (**B**) a layer chicken (Lohmann) of 27 weeks (flock 2-L) showing slight turbidity on the air sac (red circle), (**C**) a breeder quail (Baladi breed), 77 days of age (Flock 3-Q-BR), showing fibrinous air sacculitis (black arrow), (**D**) a 37-day-old turkey Poults (B6 breed (Flock 4-TR) showing caseous exudate in opened infraorbital sinus (blue circle).

Throughout the necropsy, wide ranges of gross lesions were identified in the flocks of various species of examined birds (Figure 2). In brief, flocks of chicken and quail broilers were found to have varying degrees of conjunctivitis, tracheitis, pericarditis, and perihepatitis. Furthermore, airsacculitis lesions were categorized as cloudy air sac walls, thickened air sac walls with small amounts of serofibrinous exudates, or thickened air sac walls with large accumulations of fibrinous exudates. The opened sinuses in turkey Poults were filled with mucoid to caseous exudates. Salpingitis was the most common lesion in chicken layers, aside from mild respiratory lesions. Other postmortem lesions caused by complicated infections such as greenish intestinal contents, caseated plugs in the trachea and bronchioles of the lung, nephrosis of the kidneys, petechial hemorrhages on proventriculus papillae, and congested cecal tonsils were found. The observed postmortem lesions were recorded in Table S1a–e.

### 3.3. Bacteriological Diagnostic Scheme of MG

Out of fifty-five cultured swabs, twenty-eight yielded growths on PPLO broth after 48 h (50.9%). Following 20 days of incubation, all 55 cultured PPLO broths inoculated on PPLO agar yielded 19 positive samples (34.5%) with characteristic fried egg shape colonies (tiny, smooth circular, translucent mass with a dense raised central area) under a stereomicroscope. The highest isolation rate was 66.6% in quail breeders, followed by 40% in turkey Poults, 35% in broiler chickens, 30% in chicken layers, and 0% in quail broilers. Positive flocks on the PPLO agar (n = 19) were bio-typed with digitonin, yielding 73.6% (n = 14) of the examined characteristic digitonin-sensitive colonies. Digitonin sensitivity was 100% in quail breeders and chicken layers, 75% in turkey Poults, and 42.8% in broiler chickens (Table 2).

### 3.4. Molecular Diagnostic Scheme of MG

#### 3.4.1. Molecular Screening of mgc2 Gene of MG Using qRT-PCR

A SYBR Green qRT-PCR assay revealed that 69% (n = 38/55) of the non-cultured swabs were positive for *mgc2* gene detection, whereas 89% (n = 49/55) of the tested swabs from 48 h cultured PPLO broth were positive.

#### 3.4.2. Molecular Screening of mgc2 Gene of MG Using PCR

Among the 55 swabbed flocks, 49% (n = 27/55) were positive via the PCR direct amplification of an 824 bp fragment of the *mgc2* gene from non-cultured swabs, 69% (n = 38/55) from 48 h cultured swabs in the PPLO broth, and 20% from purified characteristic colonies (n = 11/55).

### 3.5. Statistical Analysis

**Figure 3:**
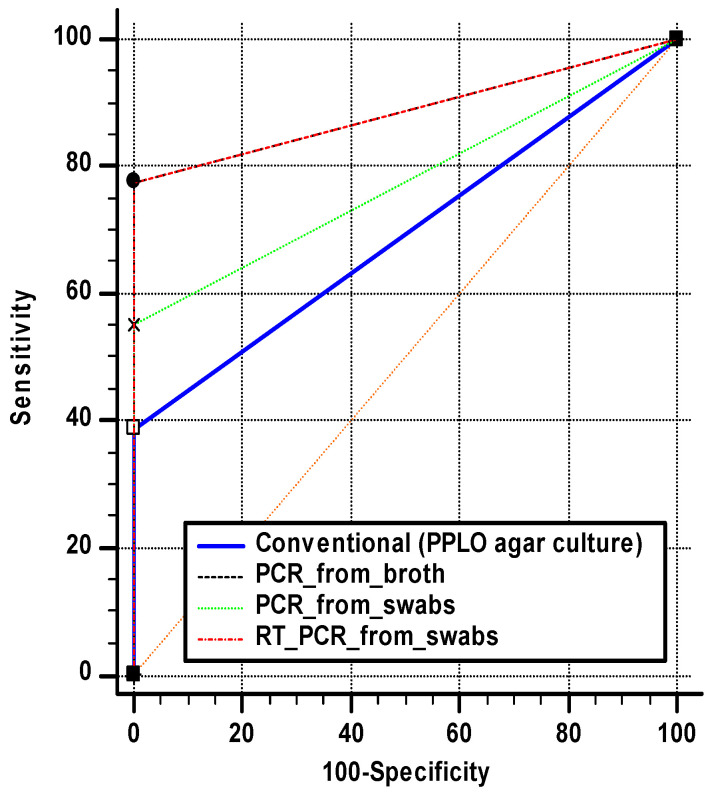
Sensitivity and specificity of four diagnostic schemes (qRT PCR from swabs, PCR from swabs, PCR from broth, and conventional culturing) for MG based on qRT PCR from broth as the gold standard test.

Based on qRT PCR from the broth as a gold standard scheme, both PCR from broth and qRT PCR from swabs exhibited the highest sensitivity and area under the curve (AUC) at 77.55% and 0.888 (*p* < 0.0001), respectively, resulting in an accuracy of 80.00%. The PCR from the swabs test showed a slightly lower sensitivity and AUC of 55.10% and 0.776 (*p* < 0.0001), respectively, while conventional culturing had the lowest sensitivity of 38.78% and an accuracy of 45.45%, as seen in Table 3. Moreover, all diagnostic schemes had 100% specificity, as seen in Figure 3. The results of Cohen’s kappa test indicate moderate agreement between qRT PCR from broth and both PCR from broth and qRT PCR from swabs, with a kappa value of 42.98%.

### 3.6. Molecular Screening of MG in Six Housekeeping Genes

In order to investigate a proper molecular target in the MG genome with superior diagnostic value, DNA extracts from 48 h of incubated PPLO broth culture of all examined flocks were screened using *mgc2*, *mraW*, *atpG*, *ugpA*, *DUF31196*, and *IgT* gene-specific primers, with PCR-amplified fragments spanning 824, 843, 822, 1071, 740, and 668 bp, respectively. The positivity rates of MG detection were 69, 65.4, 63.6, 60, 49, and 34.5%, respectively. The *mgc2* gene had the highest positivity rate, while the *IgT* gene had the lowest. The six primer sets failed to detect MG infection in the two tested quail broiler flocks. Furthermore, one tested chicken broiler flock and another chicken layer flock tested negative for *mgc2* but both were positive for *mraW*. The broiler flock 7-B was negative for *mgc2* but positive for *atpG*.

### 3.7. Sequencing and Phylogenetic Analysis

Thirty-five PCR products representing eleven MG-infected poultry flocks were partially sequenced for the six gene loci (*mgc2*, *ugpA*, *mraW*, *lgT*, *DUF31196,* and *atpG*), representing three broiler chickens, three turkey Poults, three chicken layers, and two quail breeder flocks. The accession numbers of the obtained sequences are listed in Table 4.

- mgc2

Maximum likelihood phylogenetic analysis was performed on a truncated 486 of 824 bp amplified from the *mgc2* gene of 11 different strains parallel to 16 MG reference strains (Figure S1). Seven out of the eleven analyzed strains (OP660877, OP660878, OP660880, OP660881, OP660883, OP660884, and OP660887) were grouped in a clade together with the f99 and F strains. Six of the seven strains, Egypt/MG/CH/3, 8, 22, and 26/22, Egypt/MG/TR/47/22, and Egypt/MG/QA/55/22, have 100% nucleotide identity with each other and the f99 strain, but only 99.4% with the F strain. The Egypt/MG/TR/41/22 strain shares 99.8% nucleotide identity with the other six strains and 99.2% with the F strain. Egypt/MG/CH/15, 32/22, Egypt/MG/QA/53/22, and Egypt/MG/TR/44/22, with accession numbers of OP660879, OP660882, OP660885, and OP660886, were genetically distinct from the f99 and F strains. Both Egypt/MG/CH/32/22 and Egypt/MG/TR/44/22 are the most closely related to each other, with 95.1% nucleotide identity. Egypt/MG/CH/15/22 and Egypt/MG/QA/53/2 were nearly identical divergent sequences, with 99% nucleotide identity to each other, and were clustered in distinct sub-clades with 92.5% nucleotide identity to the F strain.

- ugpA

The truncated 690 of 843 bp amplified from the *ugpA* gene of nine different strains were subjected to maximum likelihood phylogenetic analysis, alongside twenty-two other MG strains (Figure S2). Except for Egypt/MG/CH/8/22, all the amplified *ugpA* sequence fragments for the different MG strains were clustered within the same clade together with f99 and F strains, with ≥99.9% nucleotide identity. However, Egypt/MG/CH/8/22 was clustered in another branch, with 96.5–96.7% nucleotide identity to the other sequenced eight strains, as well as F and f99 reference strains.

- mraW

The truncated 626 of 822 bp amplified from the *mraW* gene of eight different strains were subjected to maximum likelihood phylogenetic analysis with twenty-three other MG reference strains. All the amplified and partially sequenced *mraW* DNA fragments were placed in the same cluster and were identical to the three vaccine MG strains, F, f99, and ts-11, with approximately 99–100% nucleotide identity (Figure S3).

- atpG

Maximum likelihood phylogenetic analysis was performed on a truncated 584 of 668 nucleotides amplified from the *atpG* gene of four different strains, along with twenty-three other MG reference strains. Three of the four strains (Egypt/MG/CH/8/22, Egypt/MG/CH/22/22, and Egypt/MG/TR/41/22) had 100% nucleotide identity with the MG vaccine strains f99 and F. The three strains share 98.1% nucleotide identity with the Egypt/Mycoplasma gallisepticum/CH/15/22 strain, which, like *mgc2*, was found in a distinct subclade with 99.5% nucleotide identity with the S6-MG strain (Figure S4).

- IgT

Maximum likelihood phylogenetic analysis was performed on a truncated 547 of 1060 nucleotides amplified from the *IgT* gene of two different strains, with twenty-two MG reference strains. With 100% nucleotide identity, the two successfully amplified and partially sequenced *IgT* fragments from Egypt/MG/CH/8/22 and Egypt/MG/TR/41/22 MG strains were clustered together with the two vaccines and the f99 and F strains (Figure S5).

- DUF31196

A truncated 631 of 740 nucleotides amplified from the *DUF31196* gene of two different strains was subjected to maximum likelihood phylogenetic analysis, with twenty-one MG reference strains. The two *DUF31196* fragments from Egypt/MG/TR/44/2022 and Egypt/MG/QA/53/2022 share 99.8% nucleotide identity with each other. Egypt/MG/TR/44/2022 has 100% nucleotide identity with the two vaccines and the f99 and F strains, whereas the quail strain Egypt/MG/QA/53/2022 shares 99.8% nucleotide identity with the two MG vaccines and the f99 and F strains (Figure S6).

### 3.8. Protein Alignment Analysis

Both point and deletion mutations were detected among the sequenced strains in comparison to the reference MG vaccine F strain (Table 5). The analysis of the *mgc2* protein sequence revealed 18 substitutions in the amino acid sequences of isolates Egypt/MG/QA/53/2022 and Egypt/MG/CH/15/22. Additionally, Egypt/MG/TR/44/2022 possesses 16 amino acid substitutions. Interestingly, there was one amino acid deletion in position 207 in both Egypt/MG/QA/53/2022 and Egypt/MG/CH/15/22. Concerning the *mraW* amino acid analysis, there were four amino acid substitutions in Egypt/MG/CH/22/22. Furthermore, only one amino acid substitution was recorded in Egypt/MG/CH/22/22 and Egypt/MG/QA/53/2022 at position 137 (E to A). The *atpG* protein sequence analysis showed that the genetic diversity existed in Egypt/MG/CH/15/22 with six amino acid substitutions; whereas, based on *ugpA* protein sequence analysis, there were three amino acid substitutions in Egypt/MG/CH/8/22.

## 4. Discussion

*Mycoplasma gallisepticum* is the most pathogenic species in the genus Mycoplasma of the Mycoplasmataceae family, causing significant economic losses [27]. The significant economic losses caused by MG include poor performance, low growth rate, mortalities, drop in egg production, carcass condemnation, and medication and vaccination costs for disease prevention and control [28]. An early and rapid diagnosis of MG is critical for disease management. For MG detection, various techniques such as isolation and cultivation, serology, and molecular identification can be applied. In the present study, to elucidate the best straightforward scheme for the MG strain analysis, different conventional and molecular approaches for MG infection detection in commercial poultry farms were evaluated. Here, this study focused on bird flocks, especially broiler chickens, which demonstrated varying degrees of respiratory distress, nervous signs, greenish diarrhea, whitish diarrhea, and variable mortalities, as recorded by Megahed et al. [29]. Accordingly, the postmortem examination of the investigated flocks revealed various extents of airsacculitis, pericarditis, and perihepatitis, as mentioned by Bharathi et al. and Emam et al. [30,31]. Furthermore, nephrosis, septicemia, caseated plugs in tracheal bifurcation, and hemorrhage on proventriculus were also recorded by Roussan et al. [32], which were suggestive of the coexistence of various ranges of respiratory pathogens [33]. Additionally, environmental stressors and respiratory reactions of some live vaccines may be included. The examined turkey flocks showed unilateral or bilateral swelling in the infra-orbital sinuses with conjunctivitis and respiratory manifestation. The previous were stated earlier by [27] in turkeys infected with MG in India. The quail breeder flock birds suffered from respiratory manifestation, mortality, and a decline in egg production [34]. A postmortem examination of quail flocks revealed fibrinous airsacculitis [35].

Although conventional culturing is considered the gold standard for MG diagnosis, this method is time-consuming and allows fastidious microorganisms to develop. In the present work, the MG culturing rate based on mycoplasma colony morphology on a PPLO agar was only 34.5%, which was lower than that reported by Heleili et al. [36] at a rate of 60.33% and by Marouf et al. [12] at a rate of 62%. The lower recovery rate of the MG may be attributed to the use of Frey’s medium as culture media compared with the PPLO agar media used in this study. A wide distribution of MG among the different investigated species suggests that these flocks did not consider the optimum biosecurity and hygienic measures in their management procedures [34]. The conventional culture techniques were laborious, costly, and time-consuming. Further, the isolation could be impaired by the overgrowth of non-pathogenic *Mycoplasma* species, such as *M. gallinarum* and *M. gallinaceum,* or other faster-growing bacteria and fungi [37].

PCR is an alternative method to the conventional culturing of MG [4], delivering accurate results in much less time [38]. The *mgc2* gene encodes the cytoskeleton surface protein cytadhesin, which is responsible for MG attachment to the host cells. Many authors and the WHO Terrestrial Manual have previously recommended it as an MG-specific molecular marker [7,37]. In the present study, the *mgc2* gene was used initially as an MG molecular target using three different molecular approaches in order to improve the MG diagnostic scheme. The approaches included direct qRT-PCR and PCR from clinical swabs, qRT-PCR and PCR from 48 h of cultured PPLO broth beside the positive colony on a PPLO agar. The positivity rate of MG detection using *mgc2* gene amplification from cultured PPLO broth (69%) was higher than direct amplification from clinical swabs (49%) and positive colonies on a PPLO agar (20%). From our results, the positivity rate in the cultured PPLO broth was higher than clinical swabs, as recorded by [39]. This may be attributed to the enrichment of MG growth in the PPLO broth, which improved the organism detection limit and the lower existence of the PCR inhibitors in PPLO broth compared with tissues.

In a trial to improve the detection sensitivity, qRT-PCR was used as another alternative technique to conventional PCR using the same amplification target [40]. Compared with qRT-PCR from cultured broth, both PCR from broth and qRT-PCR from swabs exhibited the highest sensitivity and area under the curve (AUC) at 77.55% and 0.888 (*p* < 0.0001), respectively, resulting in an accuracy of 80.00%. The results of the PCR from swabs showed a slightly lower sensitivity and AUC of 55.10% and 0.776 (*p* < 0.0001) respectively. The conventional testing had the lowest sensitivity of 38.78% and an accuracy of 45.45%. The statistical data supporting the higher sensitivity of the quantitative real-time PCR system yielded a positivity rate of 89% in the cultured PPLO broth and 69% in the non-cultured clinical swabs [41]. As evidence of the sensitivity of qRT-PCR, the quail broiler flocks were negative when tested by culture and conventional PCR; however, they were positive via using the qRT-PCR diagnostic scheme. Interestingly, all applied tests had 100% specificity, indicating the ability of these tests to avoid false positive data. These findings are supported by Cohen’s kappa test, indicating moderate agreement between the qRT PCR from the broth test and both PCR from broth and RT PCR from swabs, with a kappa value of 42.98%.

The next step was to define the best molecular target for MG detection that can be used subsequently in the pathogen detection, even in a low copy number, as well as strain classification and phylogenetic characterization. As a result, six MG housekeeping genes (*mgc2*, *mraW*, *atpG*, *ugpA*, *DUF31196,* and *lgT* genes) were investigated in 48 h of incubated cultured PPLO broth. The positivity rates were 69, 65.4, 63.6, 60, 49, and 34.5%, respectively. Accordingly, PCR based on the *mgc2* gene had the highest positivity 69% (38∕55), which agreed with that reported by Heleili et al. and Kamble et al. [36,42]. Additionally, García, et al. [37] reported that the *mgc2*-dependent PCR had a faster turnaround time than other PCR methods and was the method of choice. In our study, the amplification of *mgc2* failed in two flocks that were positive for *mraW* detection. Since the *mraW*, 16S ribosomal gene sequences are highly conserved within the bacteria and might cross-react with other bacterial species and cause false-positive results [43]. In addition, the same two flocks were consistently positive using *atpG* gene amplification, confirming the MG infection in those particular two flocks, as the *atpG* is a housekeeping gene [16,23,44]. Further, these two flocks were seen as positive by qRT-PCR in our study. The previous study confirmed the necessity of using more than one target in the process of MG detection using the PCR technique rather than only *mgc2* [7,45]. The variation in the detection levels among the six targeted genes could be attributed to many factors, including mutations in the primer sites and how many times the gene repeated within the MG genome.

To date, the best strategies for mycoplasma eradication were based on the maintenance of *mycoplasma*-free flocks. This can be achieved through the adoption of biosecurity measures [7] and the application of live vaccines as alternative control tools where eradication is too difficult [15]. Consequently, with the increase in vaccine utilization, powerful tools to capture the source of infection and differentiate the circulating field isolates from vaccine strains are needed. Thirty-five PCR products representing eleven infected poultry flocks of different species targeting six gene loci (*mgc2*, *ugpA*, *mraW*, *lgT*, *DUF31196,* and *atpG*) were partially sequenced. In addition to molecular identification, the gene target sequence analysis of the *mgc2* was also widely used [12,29,46]. The tree comparison revealed the limitation of the phylogeny of the *mraW*, *IgT,* and *DUF31196* in the classification of the MG-studied isolates into different clusters, probably due to their conservative character [47]. However, *mgc2*, *ugpA,* and *atpG* could phylogenetically discriminate the studied isolates into different clades. De la Cruz and colleagues recommend the use of the *mgc2* gene as a reliable phylogenetic, phylodynamic, and phylogeographic marker in global phylogenetic studies [48]. The phylogenetic analysis based on the *mgc2* gene sequence revealed that eleven Egyptian MG isolates were classified into two groups while in a previous study, the sequence analysis of the *mgc2* gene clustered Italian MG isolates into six main types [7]. Here, the strains (OP660877, OP660878, OP660880, OP660881, OP660884, and OP660887) were completely identical to the MG strain f99, which could explain the vaccine contaminating the examined flocks due to bad biosecurity measures. Egypt/MG/CH/15/22 and Egypt/MG/QA/53/2 were clustered in distinct subclades with 92.5% nucleotide identity to the F strain. The two strains were the closest neighbors for other pathogenic MG isolates from Thailand [6] and Egypt. The same strain Egypt/MG/CH/15/22 was separately clustered in different branches from the vaccine strains in the instance of *atpG* phylogeny together with the S6 strain. The latter is one of the most virulent field strains causing severe airsacculitis, immune suppression, and a drop in egg production of up to 50% [49,50]. However, the *ugpA* phylogeny did not discriminate the same strain, Egypt/MG/CH/15/22, from the vaccine strains but placed the Egypt/MG/CH/8/22 strain in another branch together with the virulent thoroughly investigated S6 strain.

Mycoplasma is a rapidly evolving organism that has lost many of the repair mechanisms found in other bacterial pathogens [51]. The genome evolution of MG leads to some consequences, such as evasion of the host immune system and switching to new host species [52]. According to protein alignment analysis, *mgc2* has the highest rate of amino acid substitutions compared with the other analyzed proteins. Interestingly, these amino acid substitutions were clearly demonstrated in the sequences of the three isolated strains Egypt/MG/CH/15/22, Egypt/MG/QA/53/22, and Egypt/MG/TR/44/22. It is worth mentioning that the three strains descended from three distinct species: chicken, turkey, and quail. Therefore, more research is needed to link the observed amino acid substitutions to the host shift process to new species as a host–pathogen coevolution process, as well as to fully comprehend variations in MG antigenicity and pathogenicity. As a result, efforts to mitigate the negative effects of MG on the poultry industry should be increased by implementing biosafety controls and effective vaccines.

## 5. Conclusions

In conclusion, qRT-PCR from incubated PPLO broth for 48 **h** is the best scheme for precise, sensitive, and reproducible MG detection. Furthermore, comparative phylogenetic analysis revealed that both *mgc2* and *atpG* genes sequenced MG isolates into distinct clades with the greatest discriminative power and, as a result, the highest positivity rate of MG in the field samples.

## Figures and Tables

**Table 1:** Specific primers used for the identification of MG in this study.

Gene	Primer Sequence (5 to 3)		Product Size (bp)	Reference
	Forward	Reverse		
*ugpA*	CGTAAGAATAAGCCGTATAAAGTTCC	GGTTAAGATTTGGGTGCCATTAG	843	[23]
*atpG*	CACACTTTGGATTCAATTAAACAACC	GCAATGAAKATGCTTTCAACCG	668	
*DUF31196*	GGRTAAGAAGGATAATAATCTTGCAT	TTGTGGTTAGTGGGGATAATGAA	740	
*mraW*	GGTTTGGCGGTCATAGTTAC	CAAGGACGAATAGTTTGGCTG	822	
*lgT*	CAGGCAATCATACAATAAACGATAG	CATCAGCATAAAARAACATTTCAGAG	1071	
Mgc2*	TAA ACC CAC CTC CAG CTT TAT TTC C	CGC AAT TTG GTC CTA ATC CCCAAC A	824	[15]
Mgc2**	GCT GCA CTA AAT GAT ACG TCA AA	CTA GAG GGT TGG ACA GTT ATG		[24]

Mgc2* = for regular PCR, Mgc2** = for qRT-PCR.

**Table 2:** The results of conventional culturing and digitonin biotyping.

Species	Number of Flocks	Conventional Culturing				Digitonin Bio Typing	
		48 h Incubated PPLO Broth		PPLO Agar			
		positive	%	positive	%	positive	%
Broiler chickens	20	13	65	7	35	3	42.8
Turkey Poults	10	5	50	4	40	3	75
Quail broilers	2	0	0	0	0	0	0
Chicken layers	20	8	40	6	30	6	100
Quail breeders	3	2	66.6	2	66.6	2	100
total	55	28	50.9	19	34.5	14	73.6

**Table 3:** Comparative evaluation of all the diagnostic schemes.

Items	Conventional	PCR from Broth	PCR from Swabs	qRT PCR from Swabs
True positive	19 (34.54)	38 (69.09)	27 (49.09)	38 (69.09)
False positive	0 (0.00)	0 (0.00)	0 (0.00)	0 (0.00)
True negative	6 (10.91)	6 (10.91)	6 (10.91)	6 (10.91)
False negative	30 (54.55)	11 (20.00)	22 (40.00)	11 (20.00)
Sensitivity	38.78 (25.2–53.8)	77.55 (63.4–88.2)	55.10 (40.2–69.3)	77.55 (63.4–88.2)
Specificity	100.00 (54.1–100.0)	100.00 (54.1–100.0)	100.00 (54.1–100.0)	100.00 (54.1–100.0)
AC	45.45	80	60	80
PPV	100.0 (54.1–100)	100.0 (54.1–100)	100.00 (54.1–100.0)	100.0 (54.1–100)
NPV	16.7 (13.8–20.0)	35.3 (24.5–47.9)	21.4 (16.7–27.1)	35.3 (24.5–47.9)
Kappa value	12.14	42.98	21.12	42.98
AUC	0.694 (0.555–0.811)	0.888 (0.774–0.957)	0.776 (0.643–0.877)	0.888 (0.774–0.957)
*p*-value	<0.0001	<0.0001	<0.0001	<0.0001

AC, accuracy; PPV, positive prediction value; NPV, negative prediction value; AUC, area under the curve.

**Table 4:** Description of MG strains phylogeniticaly analyzed in this study and their GenBank accession numbers.

Strain Name	Flock Code	Host Origin	Locality	Accession Number					
				*mgc2*	*ugpA*	*mraW*	*lgT*	*DUF311-96*	*atpG*
Egypt/Mycoplasma gallisepticum/CH/3/22	3-B	Chicken broiler	Sharkia	OP660877	OP660865	OP660888	NS	NS	NS
Egypt/Mycoplasma gallisepticum/CH/8/22	8-B	Chicken broiler	Sharkia	OP660878	OP660866	OP660889	OP660873	NS	OP660861
Egypt/Mycoplasma gallisepticum/CH/15/22	15-B	Chicken broiler	South Sinai	OP660879	OP660867	NS	NS	NS	OP660862
Egypt/Mycoplasma gallisepticum/CH/22/22	2-L	Chicken layer	Sharkia	OP660880	OR125944	OP660890	NS	NS	OP660863
Egypt/Mycoplasma gallisepticum/CH/26/22	6-L	Chicken layer	Sharkia	OP660881	OP660868	OP660891	NS	NS	NS
Egypt/Mycoplasma gallisepticum/CH/32/22	12-L	Chicken breeder	Port Said	OP660882	OP660869	OP660892	NS	NS	NS
Egypt/Mycoplasma gallisepticum/TR/41/22	1-TR	Turkey Poults	Sharkia	OP660883	NS	OP660893	OP660874	NS	OP660864
Egypt/Mycoplasma gallisepticum/TR/47/22	7-TR	Turkey Poults	Ismailia	OP660884	OP660870	OP660894	NS	NS	NS
Egypt/Mycoplasma gallisepticum/TR/44/22	4-TR	Turkey Poults	Sharkia	OP660885	OP660871	NS	NS	OP660875	NS
Egypt/Mycoplasma gallisepticum/QA/53/22	3-Q-BR	Quail breeder	Dakahlia	OP660886	OP660872	OP660895	NS	OP660876	NS
Egypt/Mycoplasma gallisepticum/QA/55/22	5-Q-BR	Quail breeder	Sharkia	OP660887	NS	NS	NS	NS	NS

NS = not sequenced.

**Table 5:** Amino acid substitutions based on the *mgc2* amino acid sequence.

Position	99	108	110	119	127	131	154	173	185	190	194	202	205	212	215	217	219	221	224	229	231	232	233	235	236	239	240	241	243	245	246	251
* Consensus	Q	S	T	V	D	V	L	H	P	M	H	N	P	M	R	N	P	Q	N	R	G	F	R	Q	P	G	V	S	G	K	A	N
CH/15/22	E	P	A	T	G	A	P	Q	-	I	Q	.	.	I	-	-	L	-	-	-	-	-	-	-	-	-	A	P	-	-	T	-
QA/53/22	E	P	A	T	G	A	P	Q	S	-	Q	K	S	I	-	-	L.	-	-	-	-	-	-	-	-	-	A	P	-	-	-	-
TR/41/22	-	-	-	-	-	-	-	-	-	-	-	-	-	-	-	-	-	-	-	K	C	-	-	-	-	-	-	-	-	-	-	-
TR/44/22	H	-	A	A	-	-	-	-	-	-	-	-	-	-	T	F	-	L	I	-	-	L	T	P	A	A	.	T	E	N	-	H
CH/32/22	-	-	-	-	-	-	-	-	-	-	-	-	-	-	-	-	-	-	-	-	-	-	-	-	-	-	A	-	-	-	-	-

* The amino acid sequence of the F strain was used as an alignment reference.

## Data Availability

Not applicable.

## Supplementary Materials

The following supporting information can be downloaded at https://www.mdpi.com/article/10.3390/pathogens12091131/s1, Figure S1: Molecular Phylogenetic analysis of the *mgc2* gene by Maximum Likelihood method. Phylogenetic tree based on truncated 486 bp of the MG *mgc2* gene sequence for 11 isolates (red color) with 16 published sequences on the GenBank. The Maximum Likelihood method based on the Jukes-Cantor model was used for the taxa analyzed. The bootstrap consensus trees inferred from 1000 replicates were conducted in MEGA6., Figure S2: Molecular Phylogenetic analysis of the *ugpA* gene by Maximum Likelihood method. Phylogenetic tree based on truncated 690 bp of the MG *ugpA* gene sequence for 9 representative isolates (red color) with other 22 published sequences on the GenBank. The Maximum Likelihood method based on the Jukes-Cantor model was used for the taxa analyzed. The bootstrap consensus trees inferred from 1000 replicates were conducted in MEGA6., Figure S3: Molecular Phylogenetic analysis of the *mraW* gene by Maximum Likelihood method. Phylogenetic tree based on truncated 626 bp of the MG *mraW* gene sequence of *MG* for eight representative isolates (red color) with other 23 published sequences on the GenBank. The Maximum Likelihood method based on the Jukes-Cantor model was used for the taxa analyzed. The bootstrap consensus trees inferred from 1000 replicates were conducted in MEGA6., Figure S4: Molecular Phylogenetic analysis of the *atpG* gene by Maximum Likelihood method. Phylogenetic tree based on truncated 584 bp in the MG *atpG* gene sequence for four representative isolates (red color) with 23 published sequences on the GenBank. The phylogenetic analysis was inferred by using the Maximum Likelihood method based on the Jukes-Cantor model for the taxa analyzed. The bootstrap consensus trees inferred from 1000 replicates were conducted in MEGA6., Figure S5: Molecular Phylogenetic analysis of the *IGT* gene by Maximum Likelihood method. Phylogenetic tree based on truncated 547 bp of the *IGT* gene sequence of MG for two representative isolates (red color) with 22 published sequences on the GenBank. The phylogenetic analysis was inferred by using the Maximum Likelihood method based on the Jukes-Cantor model for the taxa analyzed. The bootstrap consensus trees inferred from 1000 replicates were conducted in MEGA6. Figure S6: Molecular Phylogenetic analysis of the *DUF31196* gene by Maximum Likelihood method. Phylogenetic tree based on truncated 631 bp of the *DUF31196* gene sequence of MG for two representative isolates (red color) with 21 published sequences on the GenBank. The phylogenetic analysis was inferred by using the Maximum Likelihood method based on the Jukes-Cantor model for the taxa analyzed. The bootstrap consensus trees inferred from 1000 replicates were conducted in MEGA6; Table S1a: Description of MG infected chicken broiler flocks investigated in this study, Table S1b: Description of MG infected chicken layers and breeders flocks investigated in this study, Table S1c: Description of MG infected turkey Poults flocks investigated in this study, Table S1d: Description of MG infected quails flocks investigated in this study, Table S1e: Gross lesions recorded in Egyptian poultry flocks under investigation of Mycoplasma gallisepticum during 2019–2022.
