# Supplementary material for: Mycoplasmosis in Poultry: An Evaluation of Diagnostic Schemes and Molecular Analysis of Egyptian Mycoplasma gallisepticum Strains
Source: Pathogens. 2023 Sep 5;12(9):1131. doi: 10.3390/pathogens12091131 (PMC10536284; doi:10.3390/pathogens12091131)
Supplement: Supplementary file 1 [file pathogens-12-01131-s001.zip › supplementry figures.pdf]

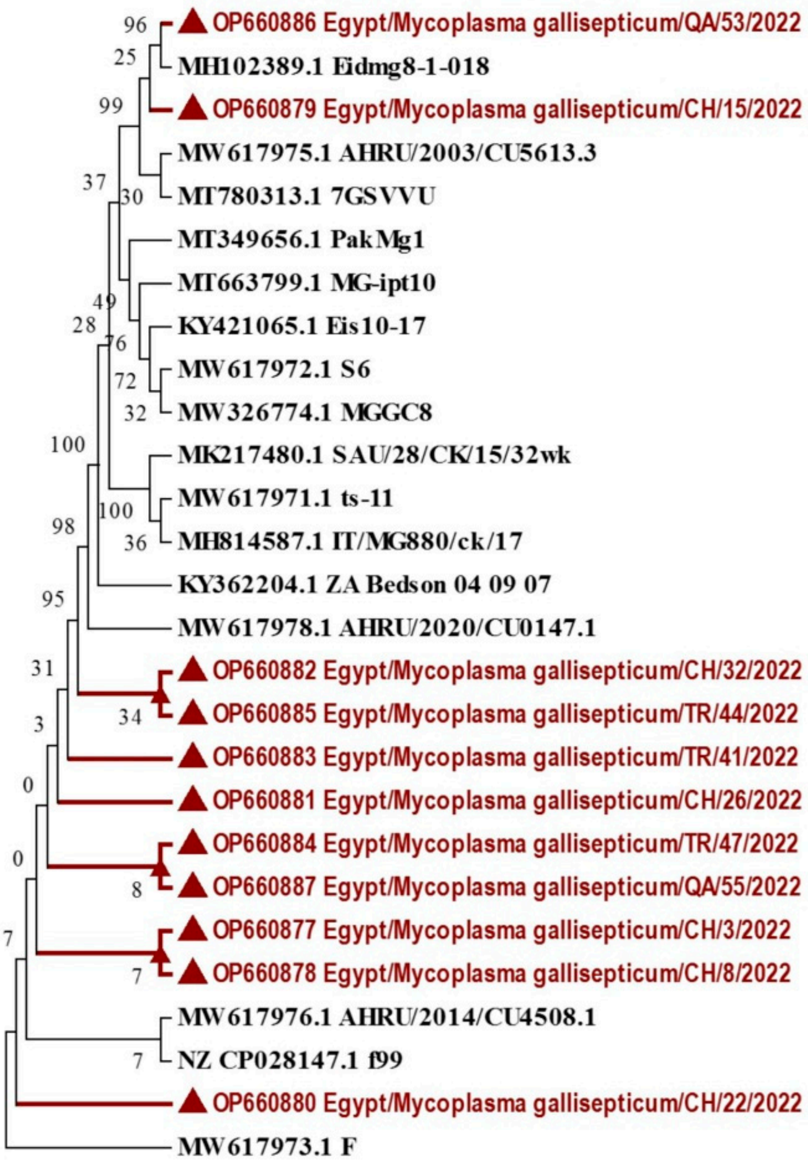

Figure S1. Molecular Phylogenetic analysis of the *mgc2* gene by Maximum Likelihood method. Phylogenetic tree based on truncated 486 bp of the MG *mgc2* gene sequence for 11 isolates (red color) with 16 published sequences on the GenBank. The Maximum Likelihood method based on the Jukes-Cantor model was used for the taxa analyzed. The bootstrap consensus trees inferred from 1000 replicates were conducted in MEGA6.

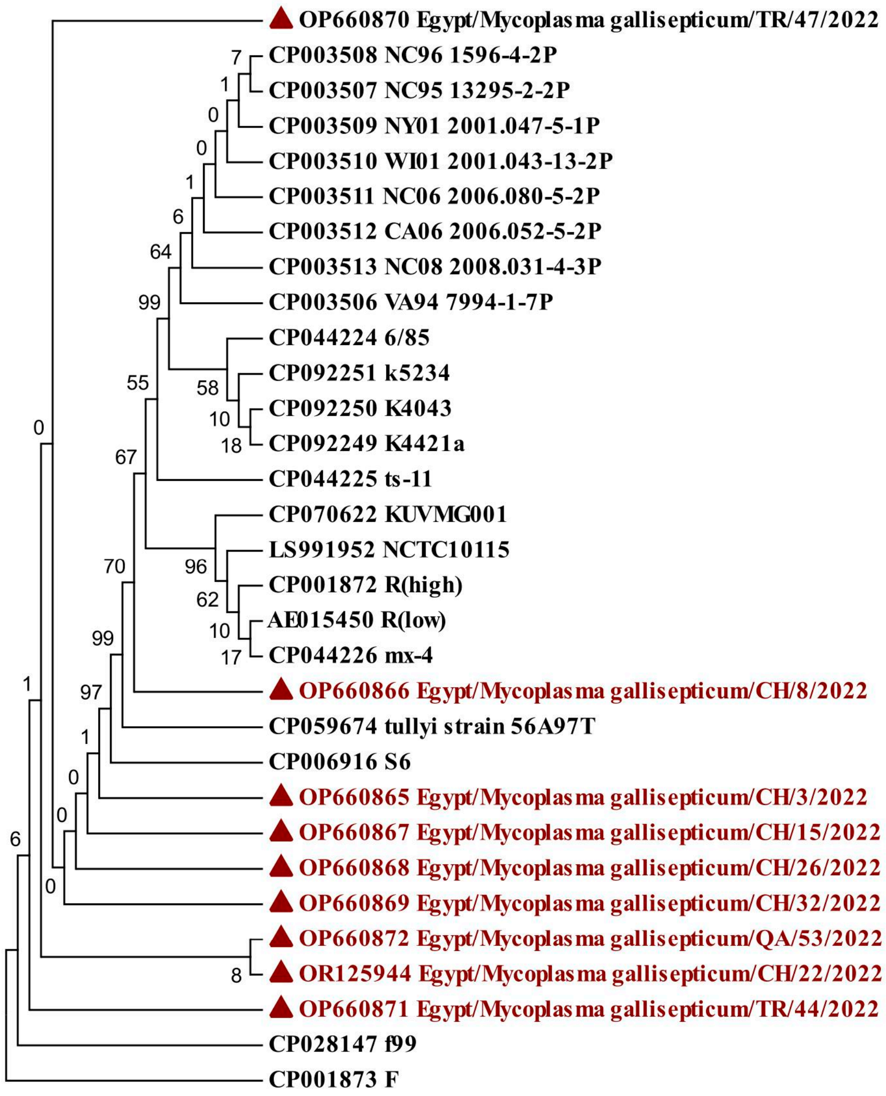

Figure S2. Molecular Phylogenetic analysis of the *ugpA* gene by Maximum Likelihood method. Phylogenetic tree based on truncated 690 bp of the MG *ugpA* gene sequence for 9 representative isolates (red color) with other 22 published sequences on the GenBank. The Maximum Likelihood method based on the Jukes-Cantor model was used for the taxa analyzed. The bootstrap consensus trees inferred from 1000 replicates were conducted in MEGA6.

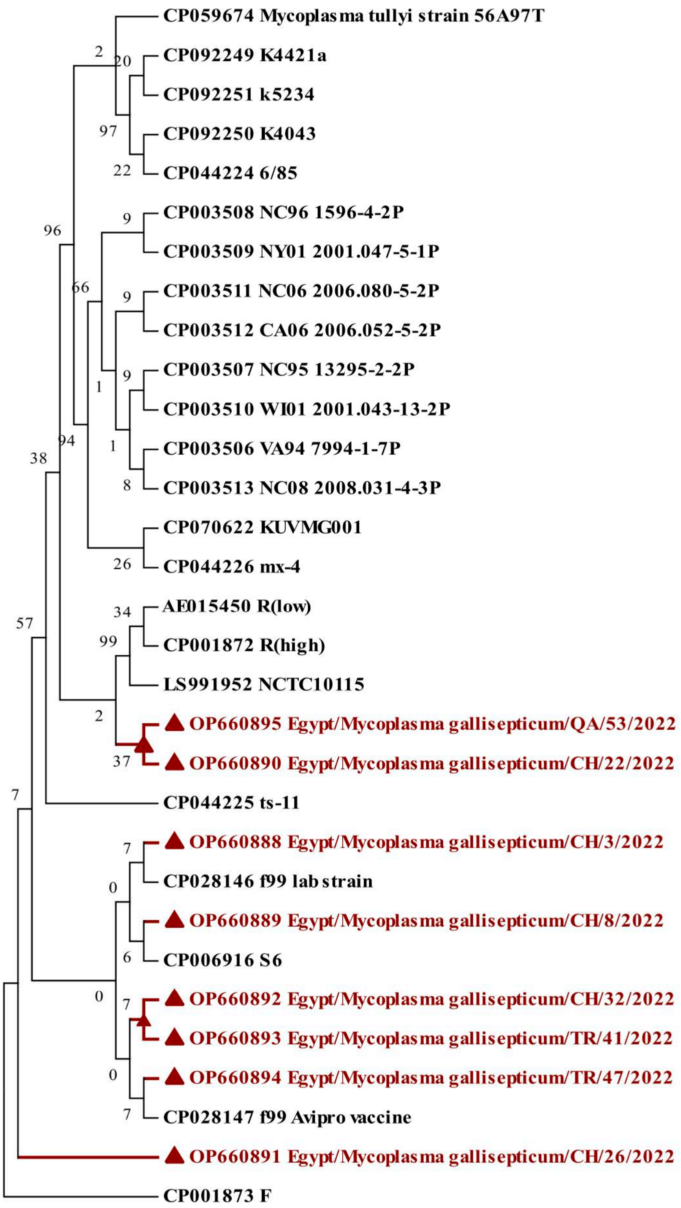

Figure S3. Molecular Phylogenetic analysis of the *mraW* gene by Maximum Likelihood method. Phylogenetic tree based on truncated 626 bp of the MG *mraW* gene sequence of *MG* for eight representative isolates (red color) with other 23 published sequences on the GenBank. The Maximum Likelihood method based on the Jukes-Cantor model was used for the taxa analyzed. The bootstrap consensus trees inferred from 1000 replicates were conducted in MEGA6.

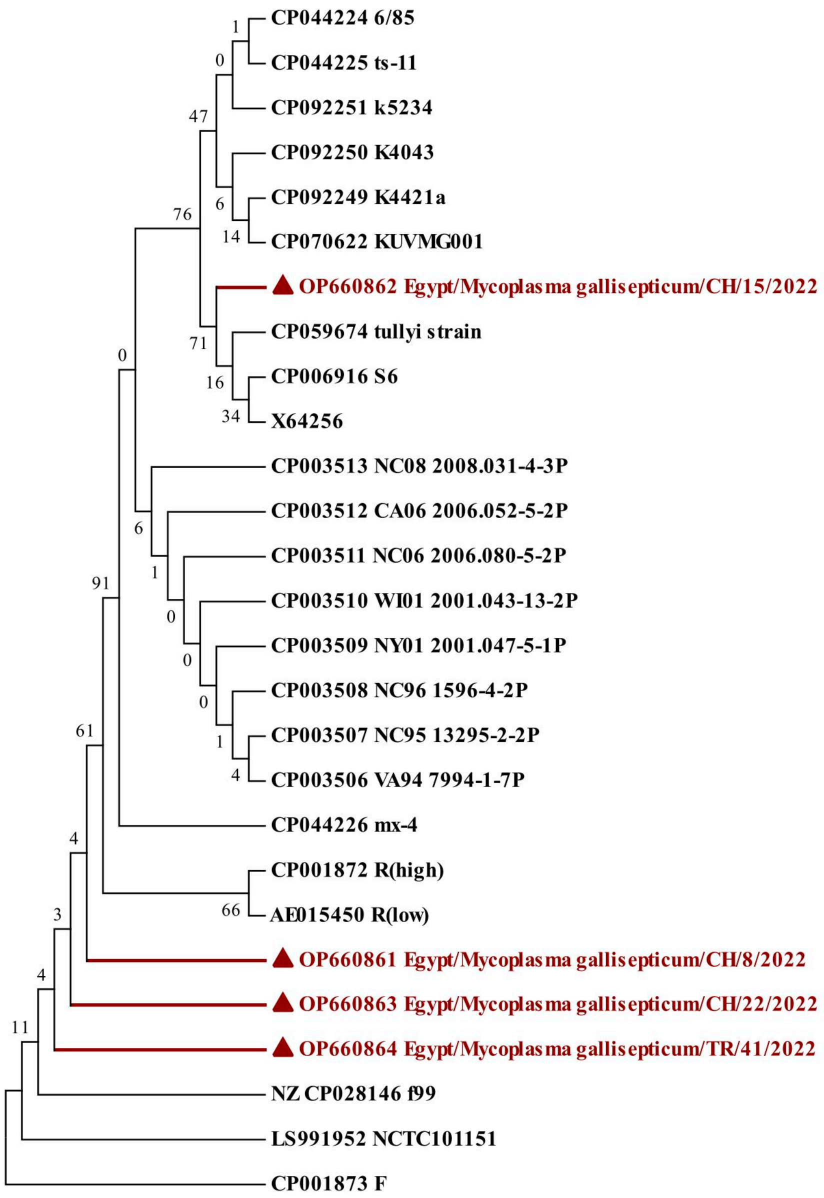

Figure S4. Molecular Phylogenetic analysis of the *atpG* gene by Maximum Likelihood method. Phylogenetic tree based on truncated 584 bp in the MG *atpG* gene sequence for four representative isolates (red color) with 23 published sequences on the GenBank. The phylogenetic analysis was inferred by using the Maximum Likelihood method based on the Jukes-Cantor model for the taxa analyzed. The bootstrap consensus trees inferred from 1000 replicates were conducted in MEGA6.

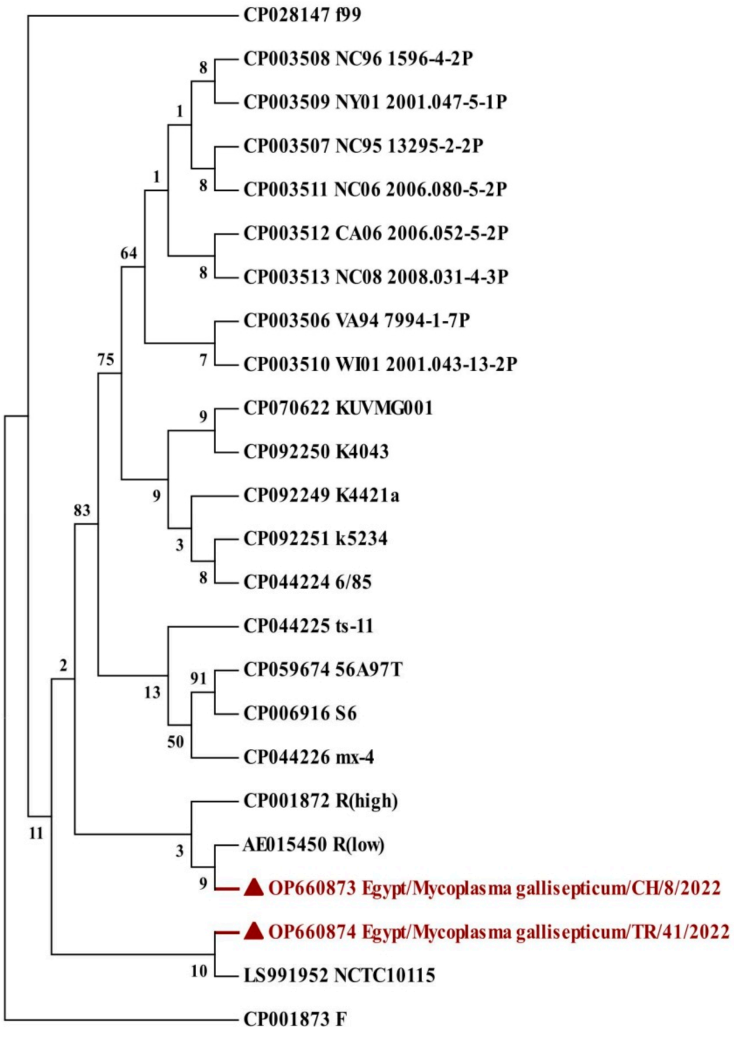

Figure S5. Molecular Phylogenetic analysis of the *IGT* gene by Maximum Likelihood method. Phylogenetic tree based on truncated 547 bp of the *IGT* gene sequence of MG for two representative isolates (red color) with 22 published sequences on the GenBank. The phylogenetic analysis was inferred by using the Maximum Likelihood method based on the Jukes-Cantor model for the taxa analyzed. The bootstrap consensus trees inferred from 1000 replicates were conducted in MEGA6.

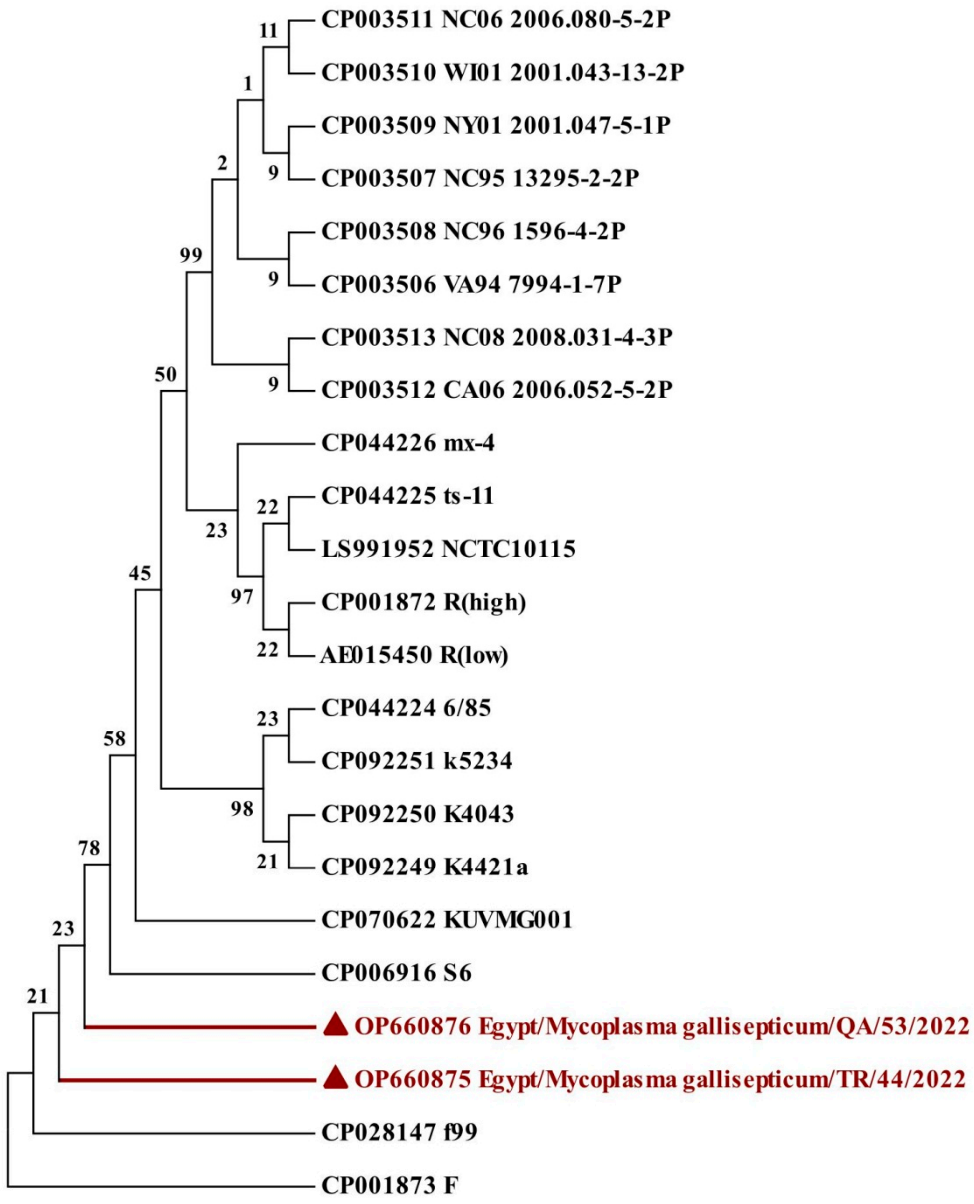

Figure S6. Molecular Phylogenetic analysis of the *DUF31196* gene by Maximum Likelihood method. Phylogenetic tree based on truncated 631 bp of the *DUF31196* gene sequence of MG for two representative isolates (red color) with 21 published sequences on the GenBank. The phylogenetic analysis was inferred by using the Maximum Likelihood method based on the Jukes-Cantor model for the taxa analyzed. The bootstrap consensus trees inferred from 1000 replicates were conducted in MEGA6.
