# Supplementary material for: Mycoplasmosis in Poultry: An Evaluation of Diagnostic Schemes and Molecular Analysis of Egyptian Mycoplasma gallisepticum Strains
Source: Pathogens. 2023 Sep 5;12(9):1131. doi: 10.3390/pathogens12091131 (PMC10536284; doi:10.3390/pathogens12091131)
Supplement: Supplementary file 1 [file pathogens-12-01131-s001.zip › Supplemmentry Tables.pdf]

Table S1 a: Description of MG infected chicken broiler flocks investigated in this study

Flock code	Breed	Total No	Age / day	Locality	MT %	Clinical examination			P.M finding		Date of collection
						Respiratory signs	Diarrhea	Other signs	P.M	Air sacs	
1-B	cobb	4000	27	Sharkia	8	+	Whitish	_	Tracheitis Kidney nephrosis + ureates in ureters	1	12- 2020
2-B	Hubbard	1800	29	Sharkia	11	+	greenish	_	Tracheitis Conjunctivitis Septicemia, Hemorrhagic cecal tonsils and greenish content in the intestine	3	05- 2019
3-B	cobb	8000	30	Sharkia	15	+	greenish	Nervous	Tracheitis Septicemia, Conjunctivitis Hemorrhagic cecal tonsils Hemorrhage on proventriculus and greenish content in the intestine	3	02- 2021
4-B	avian 48	60000	24	Sharkia	8	+	greenish	_	Tracheitis Conjunctivitis Septicemia, Hemorrhagic cecal tonsils Hemorrhage on proventriculus and greenish content in the intestine	3	06- 2021
5-B	IR	120000	22	Ismailia	20	+	Whitish	_	Tracheitis Conjunctivitis Caseated plugs in trachea and kidney nephrosis	3	12- 2020
6-B	Arbor Acres	5000	4	Dakahlia	13	+	-	enlarged abdomen	Tracheitis, Unabsorbed yolk sac and Septicemia,	2	10- 2020
7-B	Ross	6000	36	Damietta	10	+	greenish	_	Tracheitis Conjunctivitis Hemorrhagic cecal tonsils and greenish content in the intestine	3	02- 2020
8-B	Hubbard	1200	29	Sharkia	18	+	Whitish	Lameness (arthritis)	Tracheitis Conjunctivitis Caseated plugs in tracheal bifurcation, Kidney nephrosis and caseous exudates in the hock joint	3	12 - 2019
9-B	Cobb	8000	22	Sharkia	11	+	Whitish	_	Tracheitis Conjunctivitis Caseated plugs in tracheal bifurcation and Kidney nephrosis	3	03-2021
10-B	Hubbard	4700	20	Sharkia	9	+	Whitish	_	Tracheitis Conjunctivitis Caseated plugs in tracheal bifurcation and Kidney nephrosis	3	12 - 2021
11-B	Ross	9000	26	Sharkia	14	+	greenish	_	Tracheitis Conjunctivitis Hemorrhagic cecal tonsils greenish content in the intestine	3	07- 2021
12-B	IR	3800	26	Sharkia	8	+	-	_	Tracheitis friable liver, ascites and enlarged gall bladder	2	01 - 2021
13-B	Ross	1000	25	Sharkia	5	+	Whitish	_	Tracheitis Enlarged bursa, Kidney nephrosis and Hemorrhage on thigh muscles	2	02 - 2021
14-B	Baladi	24000	40	Ismailia	12	+	Whitish	_	Tracheitis Conjunctivitis Caseated plugs in tracheal bifurcation and Kidney nephrosis	3	08- 2021
15-B	Ross	12000	25	South Sinai	14	+	greenish	Nervous	Tracheitis Conjunctivitis Septicemia, hemorrhagic cecal tonsils Hemorrhage on proventriculus and greenish content in the intestine	3	12 - 2022
16-B	Baladi	16000	56	South Sinai	6	+	-	Pox lesion	Tracheitis Conjunctivitis Wart like nodules in comb and wattles and peak	3	09-2020
17-B	IR	900	19	Sharkia	3	+	Brownish	-	Tracheitis swollen intestine filled with gas with brownish content	3	01- 2020
18-B	Ross	1800	24	Sharkia	3	+	Brownish	-	Tracheitis, friable liver and enlarged gall bladder with brownish intestinal	2	02- 2021

									content		
19-B	avian 48	2500	23	Sharkia	12	+	Whitish	-	Tracheitis Enlarged yellowish bursa and kidney nephrosis	2	06- 2021
20-B	Baladi	14000	43	Port Said	10	+	-	-	Hemorrhagic Tracheitis with bloody exudate in trachea Conjunctivitis	1	07- 2020

*MT= the mortality rate was calculated along the course of the disease

B=broiler, IR=Indian River, MT=mortality, Respiratory signs= nasal discharge, sneezing, coughing and Rales && Score 0= no lesions, Score l= cloudy air sac walls,

Score 2= thickened air sac walls and small amounts of serofibrinous exudates in the air sac & Score 3= thickened air sac walls, with large accumulation of cheesy (fibrinous) exudates

Table S1 b: Description of MG infected chicken layers and breeders flocks investigated in this study

Flock code	Breed	Total No	Age / week	Locality	MT %	Clinical examination			P.M finding		
						Respiratory signs	Diarrhea	Other signs	P.M	Air sacs	
1-L	ISA Brown	8000	31	Dakahlia	1	+	whitish	Dec egg 20%	Tracheitis, kidney nephrosis	0	07- 2020
2-L	Lohmann	1000	27	Sharkia	3	+	-	Dec egg 30%	Tracheitis, Kidney nephrosis and congested ovary with ruptured ovum, Salpingitis and Egg peritonitis	1	01- 2021
3-BR	Baladi	6000	25	Sharkia	2	+	greenish	Dec egg 40%	Tracheitis, Hemorrhagic cecal tonsils, congested ovary, greenish content in the intestine and septicemia	1	12- 2019
4-L	H&N	2500	26	Sharkia	2	+	-	Dec egg 11%	Tracheitis, Caseated exudates in the oviduct	1	08- 2020
5-BR	Baladi	4000	29	Ismailia	2	+	-.	Dec egg 8%	Tracheitis and Salpingitis	1	09- 2021
6-L	H&N	8000	34	Sharkia	3	+	greenish	Dec egg 43%	Tracheitis, Septicemia, egg peritonitis, Salpingitis, greenish content in and intestine and Hemorrhagic cecal tonsils	1	08- 2020
7-BR	Baladi	9000	40	Dakahlia	2	+	greenish	Dec egg 43%	Tracheitis, Congested ovary, Egg peritonitis greenish content in intestine, septicemia, Hemorrhage on proventriculus and Hemorrhagic cecal tonsils	1	03-2020
8-L	Hy-Line	7000	37	Dakahlia	2	+	-	Dec egg 14%	Tracheitis, Egg peritonitis and caseation in oviduct	1	07- 2020
9-L	Lohmann	1800	28	Sharkia	2	+	whitish	Dec egg 18%	Tracheitis, Salpingitis Congested ovary and kidney nephrosis	1	01- 2020
10-L	H&N	9000	40	Sharkia	2	+	-	Dec egg 10%	Tracheitis, Salpingitis, Egg peritonitis with flaccid ovary	1	12- 2021
11-L	Hy-Line	6000	38	Sharkia	2	+	-	Dec egg 36% + nervous	Tracheitis, Salpingitis, Congested trachea and ovary	1	07- 2021
12-BR	Baladi	12000	31	Port Said	4	+	-	Dec eggs 26 %	Tracheitis, Salpingitis, septicemia and Egg peritonitis	1	12- 2019
13-L	Lohmann	8000	34	Sharkia	1	+	-	Dec egg 8%	Tracheitis, Congested ovary	0	08- 2021
14-L	ISA Brown	1900	23	Sharkia	3	+	-	Dec egg 40%	Hemorrhage in trachea and Salpingitis	1	08- 2021
15-BR	Baladi	6000	25	Ismailia	2	+	-	Dec egg 16%	Tracheitis, Salpingitis, Enlarged liver& spleen and egg peritonitis	1	02- 2021
16-BR	Baladi	10000	1	Ismailia	2	+	whitish	-	Tracheitis, Kidney nephrosis and visceral gout	1	04-2021
17-BR	Baladi	7000	42	Dakahlia	1	+	whitish	Dec egg 9%	Tracheitis, Salpingitis Congested ovary and kidney nephrosis	0	05-2020

18-L	Lohmann	5000	30	Dakahlia	2	+	whitish	Dec egg 16%	Tracheitis, Congested ovary, Egg peritonitis, Salpingitis and kidney nephrosis	1	02- 2020
19-L	H&N	4000	27	Sharkia	2	+	-.	Dec egg 6%	Tracheitis, Caseation in the oviduct	0	06- 2020
20-L	ISA Brown	11000	43	Sharkia	1	+	-	Dec egg 10%	Tracheitis, Caseation in oviduct and flaccid ovary	0	06- 2021

*MT= the mortality rate was calculated along the course of the disease

L=layers, BR=breeders, MT=mortality, Dec=decrease egg production, Respiratory signs= sneezing, coughing and Rales & Score 0= no lesions, Score l= cloudy air sac walls,
Score 2= thickened air sac walls and small amounts of serofibrinous exudates in the air sac & Score 3= thickened air sac walls, with large accumulation of cheesy (fibrinous) exudates

Table S1 c: Description of MG infected turkey Poults flocks investigated in this study

Flock No	Breed	Total No	Age / day	Locality	MT %	Clinical examination			P.M finding		Date of collection
						Respiratory signs	Diarrhea	Other signs	P.M	Air sacs	
1-TR	Baladi	8000	14	Sharkia	18	+	greenish	infraorbital Sinusitis	Conjunctivitis, Tracheitis, bilateral caseous exudate in sinus Septicemia, Hemorrhage on proventriculus, greenish content in the intestine and hemorrhagic cecal tonsils	3	06- 2020
2-TR	Grand maker	1800	9	Ismailia	8	+	Whitish	infraorbital sinusitis	Conjunctivitis, Tracheitis, Caseated exudate in trachea bifurcation , bilateral Mucoid exudate in sinus and kidney nephrosis	2	01- 2021
3-TR	XL	4000	65	Dakahlia	5	+	greenish	infraorbital sinusitis	Conjunctivitis, Tracheitis, bilateral caseous exudate in sinus, Septicemia, hemorrhagic cecal tonsils and greenish content in the intestine	3	11- 2021
4-TR	B6	2400	37	Sharkia	19	+	Whitish	infraorbital sinusitis	Conjunctivitis, Tracheitis, Caseated exudate in trachea bifurcation, unilateral Mucoid exudate in sinus and kidney nephrosis	1	02- 2021
5-TR	Baladi	1500	8	Ismailia	11	+	Whitish	-	Tracheitis, Caseated exudate in trachea bifurcation and kidney nephrosis	0	12-2021
6-TR	Baladi	4000	19	Sharkia	8	+	greenish	infraorbital sinusitis	Conjunctivitis, Tracheitis and bilateral Mucoid exudate in sinus, Septicemia, Hemorrhage on proventriculus, greenish content in the intestine and hemorrhagic cecal tonsils	3	11- 2020
7-TR	Grand maker	1600	33	Ismailia	25	+	Whitish	infraorbital sinusitis	Conjunctivitis, Tracheitis, caseation in tracheal bifurcation, unilateral Mucoid exudate in sinus and Nephritis	2	01-2020
8-TR	Baladi	6000	16	Sharkia	4	+	greenish	infraorbital sinusitis	Conjunctivitis, Tracheitis, Septicemia, friable liver, hemorrhagic cecal tonsils and greenish content in the intestine and bilateral caseous exudate in sinus	3	04- 2022
9-TR	Baladi	1000	90	Sharkia	8	+	-	Lameness (arthritis)	Tracheitis, Septicemia, Necrotic foci on liver and yellowish exudate in hock joints	0	08- 2021
10-TR	XL	900	78	Sharkia	10	+	Brownish	infraorbital sinusitis	Conjunctivitis, Tracheitis, hydro pericardium, enteritis and unilateral Mucoid exudate in sinus	1	06- 2021

*MT= the mortality rate was calculated along the course of the disease
TR=turkeys, MT=mortality, Respiratory signs=ocular discharge, nasal discharge, sneezing and coughing & Score 0= no lesions, Score l= cloudy air sac walls,
Score 2= thickened air sac walls and small amounts of serofibrinous exudates in the air sac & Score 3= thickened air sac walls, with large accumulation of cheesy (fibrinous) exudates

Table S1 d: Description of MG infected quails flocks investigated in this study

Flock code	Breed	Total No	Age / day	Locality	MT %	Clinical examination			P.M finding		Date of collection
						Respiratory signs	Diarrhea	Other signs	P.M	Air sacs	
1-Q-B	Baladi	1500	22	Sharkia	3	+	Whitish	-	Tracheitis, friable liver, ulcer on intestinal wall, enlarged gall bladder and kidney nephrosis	2	07- 2020
2-Q-B	Baladi	2000	25	Damietta	13	+	Greenish	-	Conjunctivitis, Tracheitis, Septicemia, Hemorrhagic cecal tonsils and greenish content in the intestine	0	02- 2021
3-Q-BR	Baladi	3000	77	Dakahlia	5	+	Greenish	Dec egg pro 30 %	Tracheitis, Egg peritonitis and greenish content in the intestine	2	02- 2022
4-Q-BR	Baladi	3500	101	Dakahlia	3	+	Whitish	Dec egg pro 10 %	Tracheitis, Egg peritonitis	3	06-2020
5-Q-BR	Baladi	2000	84	Sharkia	2	+	Whitish	Dec egg pro 20 %	Tracheitis, congested ovary	2	01- 2021

*MT= the mortality rate was calculated along the course of the disease

Q-B=quail broiler, Q-BR= quail breeder, Dec=decrease egg production, MT=mortality, Respiratory signs=ocular discharge, nasal discharge, sneezing and coughing &

Score 0= no lesions

Score l= cloudy air sac walls

Score 2= thickened air sac walls and small amounts of serofibrinous exudates in the air sacs

Score 3= thickened air sac walls, with large accumulation of cheesy (fibrinous) exudates

Table S1 e: Gross lesions recorded in Egyptian poultry flocks under investigation of *Mycoplasma gallisepticum* during 2019-2022

PM lesions	Chicken broilers	Chicken layers and breeders	Turkey Poults	Quail broilers	Quail breeders
Respiratory					
Conjunctivitis	13/20	-	8/10	1/2	-
Tracheitis	20/20	20/20	10/10	2/2	3/3
Caseation at tracheal bifurcation	6/20	-	4/10	-	-
Air sacs					
Score 0	-	5/20	2/10	1/2	-
Score 1	2/20	15/20	2/10	-	-
Score 2	5/20	-	2/10	1/2	2/3
Score 3	13/20	-	4/10	-	1/3
Sinusitis					
Mucoid exudate	-	-	5/10	-	-
Caseous exudate	-	-	3/10	-	-
Greenish content in intestine	6 /20	3/20	4/10	1/2	1/2
Kidney nephritis/nephrosis	8/20	5/20	4/10	1/2	-
Hemorrhagic cecal tonsils	6/20	3/20	4/10	1/2	-
Hemorrhage on proventriculus	3/20	1/20	2/10	-	-
Salpingitis	-	15/20	-	-	-
Egg peritonitis	-	8/20	-	-	2 /3

Score 0= no lesions

Score l= cloudy air sac walls

Score 2= thickened air sac walls and small amounts of serofibrinous exudates in the air sacs

Score 3= thickened air sac walls, with large accumulation of cheesy (fibrinous) exudates
